# Feasibility of digital contact tracing in low-income settings – pilot trial for a location-based DCT app

**DOI:** 10.1186/s12889-022-14888-x

**Published:** 2023-01-21

**Authors:** Eric Handmann, Sia Wata Camanor, Mosoka P. Fallah, Neima Candy, Davidetta Parker, André Gries, Thomas Grünewald

**Affiliations:** 1 Department for Emergency Medicine, University Hospital Leipzig, Leipzig, Germany; 2 JFK Hospital, Monrovia, Liberia; 3 National Public Health Institute of Liberia (NPHIL), Monrovia, Liberia; 4 Clinic for Infectious Diseases and Tropical Medicine and Department for Hospital and Environmental Hygiene, Klinikum Chemnitz, Chemnitz, Germany

**Keywords:** App, Digital contact tracing, Low income, GPS tracking, Location based, Public health

## Abstract

**Background:**

Data about the effectiveness of digital contact tracing are based on studies conducted in countries with predominantly high- or middle-income settings. Up to now, little research is done to identify specific problems for the implementation of such technique in low-income countries.

**Methods:**

A Bluetooth-assisted GPS location-based digital contact tracing (DCT) app was tested by 141 participants during 14 days in a hospital in Monrovia, Liberia in February 2020. The DCT app was compared to a paper-based reference system. Hits between participants and 10 designated infected participants were recorded simultaneously by both methods. Additional data about GPS and Bluetooth adherence were gathered and surveys to estimate battery consumption and app adherence were conducted. DCT apps accuracy was evaluated in different settings.

**Results:**

GPS coordinates from 101/141 (71.6%) participants were received. The number of hours recorded by the participants during the study period, *true Hours Recorded* (*tHR*), was 496.3 h (1.1% of *maximum Hours recordable)* during the study period. With the paper-based method 1075 hits and with the DCT app five hits of designated infected participants with other participants have been listed. Differences between true and maximum recording times were due to failed permission settings (45%), data transmission issues (11.3%), of the participants 10.1% switched off GPS and 32.5% experienced other technical or compliance problems.

In buildings, use of Bluetooth increased the accuracy of the DCT app (GPS + BT 22.9 m ± 21.6 SD vs. GPS 60.9 m ± 34.7 SD; *p* = 0.004). GPS accuracy in public transportation was 10.3 m ± 10.05 SD with a significant (*p* = 0.007) correlation between precision and phone brand. GPS resolution outdoors was 10.4 m ± 4.2 SD.

**Conclusion:**

In our study several limitations of the DCT together with the impairment of GPS accuracy in urban settings impede the solely use of a DCT app. It could be feasible as a supplement to traditional manual contact tracing.

DKRS, DRKS00029327. Registered 20 June 2020 - Retrospectively registered.

**Supplementary Information:**

The online version contains supplementary material available at 10.1186/s12889-022-14888-x.

## Background

Contact tracing was an essential element of the containment strategy of the 2014–2016 Ebola epidemic in West Africa. However, postepidemic analysis has revealed many limitations and challenges associated with manual contact tracing (MCT) [1]. These limitations primarily include poor scalability, time-consuming in-person interviews with infected patients and difficulty in contacting, informing and tracking exposed contacts. Additionally, recall errors, undetected exposures in public settings, transcription errors and time delays between contact identification and the instruction to quarantine further impede the effectiveness of contact tracing [1, 2]. MCT is a time-consuming process and thus challenging in overwhelmed health systems coping with high numbers of infections.

Smartphone-based digital contact tracing (DCT) may address some of these limitations. Modeling studies [3] suggest that epidemic control can be achieved using DCT. Key enablers for the use of DCT include the continuing growth in coverage of mobile cellular networks, rapid advances in mobile technologies and the integration of mobile health tools into existing electronic health services [4]. From 2015 to 2021, the percentage of the population in the least developed countries being covered by at least 3G mobile networks has increased in 6 years from 53 to 83%. The number of mobile phone subscriptions in these countries increased from 67 to 75% in this time period [5]. To exploit this potential, the WHO has called for innovative technologies that address global health concerns in limited-resource settings [6].

During the COVID-19 pandemic, numerous high- and middle-income countries developed and deployed DCT tools. The reported success of DCT differed widely due to diverse policies for DCT (e.g., extent of privacy invasion), varying technical approaches and cultural differences [7, 8]. A review of current studies [9] identified limitations of DCT, such as dependency on a high degree of adoption and adherence, privacy concerns in the population, security vulnerability and technical constraints. Considering differences between DCT approaches and their varying performances throughout the countries, it becomes apparent that one solution cannot fit all and thus needs to be adapted to the political, economic, cultural and social characteristics of the targeted population.

The aim of this study was to evaluate a DCT app in the setting of a low-income country. Widely available techniques such as GPS and Bluetooth were used to maximize the proportion of the population meeting the technical requirements to use the DCT app. Other emerging techniques to estimate proximity (e.g., Bluetooth Low Energy - BTLE) would exclude a significant number of individuals using older smartphones [10]. The DCT app used regular Bluetooth to mitigate the deficiencies of GPS in urban settings, such as the inability of GPS to differentiate between app users on different floors. To assess its effectiveness, the DCT app was compared to a paper-based method. A secondary outcome was to identify specific restrictions for the implementation of DCT under real-world conditions of limited-resource countries.

## Methods

A field trial was designed and established for 14 days at the John F. Kennedy Medical Center in Monrovia, Liberia, in February 2020. Ethical approval was granted by the Institutional Review Board Committee of the John F. Kennedy Memorial Medical Center. The study received clearance and support from the National Public Health Institute of Liberia and the Ministry of Health of the Republic of Liberia.

Recruitment of participants took place 4 days prior to the trial by providing information about the planned trial and consecutive sampling. Study participants were employees of the JFK Hospital. Inclusion criteria comprised written informed consent, a smartphone with an Android operating system (OS) capable of GPS, Bluetooth and mobile data and the possibility to perform a paper-based reference system (pCT). The target sample size was 200. Participants who did not attend the first or third visit or presented a smartphone different from the one they registered at the kick-off meeting were excluded. Participants were provided with mobile data credit, and transportation fees were covered.

**Fig. 1:**
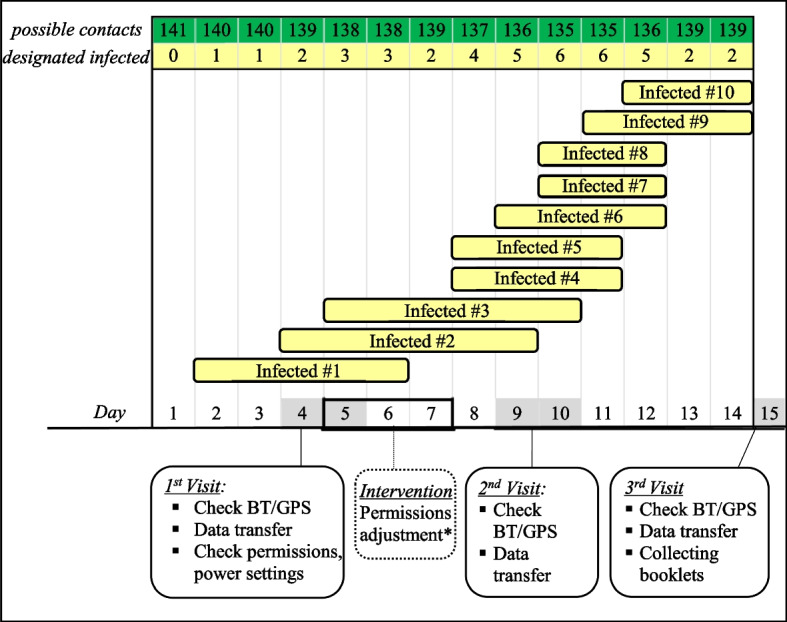
Course of study with participants successively designated as infected showing their cumulative number and the number of possible contacts for each day; the timing of the intervention and the visits with the respective tasks was mapped on the timeline. * Permission for the DCT app to use location, storage and phone (Bluetooth, mobile data) of the smartphone

The training phase was held during two kick-off meetings, where the DCT app called EBOLAPP was sideloaded via a QR code from a free cloud-based storage service and installed on the participants’ smartphones. Booklets and armlets were distributed to the participants along with further details on study procedures. After the first visit, issues with the automatic setting of permissions for the DCT app to use location, storage and phone (Bluetooth, mobile data) of the smartphone were identified. Therefore, a manual adjustment of permissions was added to the study procedure. During the study, ten participants were designated ‘infected’ for between three and 6 days (Fig. 1).

The DCT app records GPS coordinates every 30 seconds if a position change of greater than 1 meter is detected. If no position change is detected for 25 minutes, the criteria are reduced to every 5 minutes and five meters to preserve battery life. When Bluetooth detects another device, the last GPS coordinate logged is added to the movement profile to increase the frequency of logged GPS coordinates. For subjects who are subsequently identified as infected, their anonymised coordinate data are voluntarily shared to all other app users to determine a match with their own locally stored coordinate data. Coordinate data received from other app users are immediately deleted after coordinate matching occurs. The proximity thresholds measured in meters and time matching intervals for hit registration are adjustable by the administrator. If the coordinates match, the app user receives an exposure notification. For the purposes of this study, the GPS coordinate logs of all participants were sent to the server during each of the three visits. During the visits, additional data about GPS and Bluetooth adherence were gathered, and a survey to estimate battery consumption and adherence to app use was conducted. Significant exposure risk was defined to have occurred for hits with a proximity of ≤5 m for ≥2 minutes and for hits ≤1.5 m irrespective of duration.

To assess the effectiveness of the digital contact tracing app (aCT), a paper-based reference system for contact tracing (pCT) was established. To enable visual discrimination, study participants wore a yellow armlet if they were designated as infected and a green armlet if they were not. Participants listed all hits with designated infected participants with the same definitions for proximity and duration as aCT according to date and time, and infected participants did the same vice versa.

To process the proximity and duration of hits based on the DCT app GPS coordinate logs, a Javascript-based software with an identical algorithm to the DCT app was used (Additional File 1). The proximity of GPS coordinates was determined using the spherical law of cosines. After the lists of pCT hits were digitalized, they were matched with aCT hits by analyzing the data using R and SPSS to evaluate the aCT accuracy.

**Fig. 2:**
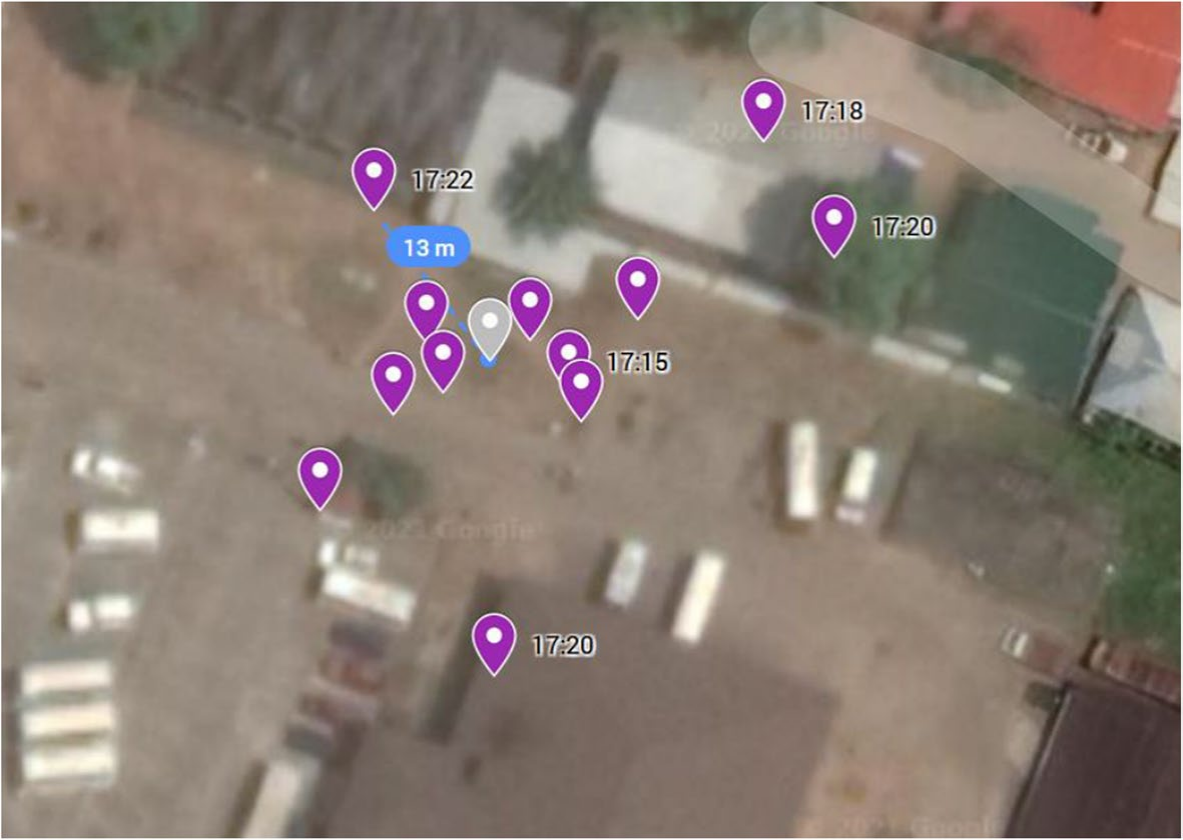
Measurement of accuracy during use in an outside urban environment. Distance of logged GPS coordinate (violet) to location of the participant (grey) in m. Time of logged GPS coordinate in hh:mm

To evaluate the DCT app accuracy in buildings, an index smartphone was positioned in the ground floor of a three-floor building for 2 min within a distance of five meters to randomly chosen participants. To assess the outdoor accuracy in an urban environment, participants were positioned at distinct locations of the city, and the precision of participants’ logged GPS coordinates was measured (Fig. 2). Moreover, participants were asked to track their commute using a car or public transport, and every logged GPS coordinate on the route was visualized and analyzed in reference to an idealized route (Additional File 2). Google Maps was used to visualize and determine the precision of GPS coordinates.

The Shapiro–Wilk test was used to check for a normal distribution. Chi-square analyses for categorical variables and the Mann–Whitney U test for continuous variables were used to analyze differences among rates, and Spearman correlation was used to assess the association between two variables. *p* values were determined for all outcomes and considered significant at < 0.05.

## Results

**Fig. 3:**
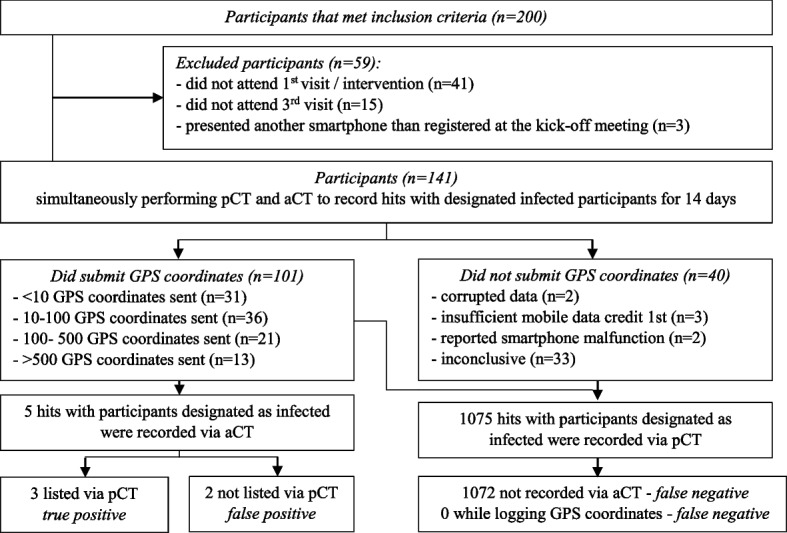
Flow of participants and results of the contingency table for effectiveness

From a total of 200 included participants, 59 were excluded (see Fig. 3 for an explanation). The median age of all 141 participants was 31 years (IQR 27–36), and 58.2% were female. The professions, distribution of phone brands and Android OS versions are displayed in Table 1, and information about the smartphone models used is added in Additional File 3. A total of 71.4% of the participants used smartphone brands where the DCT app had not been previously tested during a pilot study (Table 1). Thus, specific settings of those smartphone brands and OS were not implied during app development. Smartphones with outdated Android versions (Android 4, 5 and 6) were used by 21.6% of participants (the latest Android version during the time the study was conducted was Android 10).

**Table 1:** Baseline characteristics of participants and smartphones

*Participants characteristics*	n (%)*Total n* = 141
*Median age*, years (range)	31 (27–36)
*Women*	82 (58.2)
*Profession*	
Nurse	41 (29.1)
Medical student	35 (24.8)
Midwife	15 (10.6)
Environmental health	10 (7.1)
Physician assistant	9 (6.4)
Security	6 (4.3)
Maintenance	5 (3.5)
Social worker	4 (2.8)
Administration	3 (2.1)
Pharmacist	2 (1.4)
Cleaner	2 (1.4)
Others^a^	10 (7.1)
*Phone brand*	
Tecno	56 (39.7)
Samsung*	32 (22.7
Itel	20 (14.2)
Infinix	9 (6.4)
BLU	3 (2.1)
ZTE	3 (2.1)
Orange	2 (1.4)
HTC*	2 (1.4)
LG*	2 (1.4)
One	2 (1.4)
Xiaomi	2 (1.4)
Others^b^	7 (4.9)
unknown	1 (0.7)
*Android version*	
4	4 (2.8)
5	7 (5.0)
6	17 (12.1)
7	28 (19.9)
8	43 (30.5)
9	31 (22.0)
unknown	11 (7.8)

^a^*n* = 1: biologist, paramedic, optician, biomedical engineer, laboratory assistant, receptionist, journalist, cook, receptionist, data coordinator

^b^*n* = 1: Alcatel, Motorola*, Vivo, A3, MobiWire, Nokia*, Huawei

*previously tested Smartphone brands

GPS coordinates from 101/141 (71.6%) participants were received (Fig. 3). Of the 40 participants, from whom no GPS coordinates were received, specific reasons were identified for seven cases (Fig. 3). For 33 participants for whom no specific reason was identified, compatibility between the software and smartphone might be an explanation: 30/33 (90.9%) used brands that were not previously tested. Compared to the overall distribution of not previously tested brands (71.4%), this proportion is significantly higher (*p* = 0.015).

Of the 101 participants who transmitted GPS coordinates to the server, 31 (30.7%) sent less than ten GPS coordinates over 14 days, and 67 participants (66.3%) transmitted less than 100 GPS coordinates (Fig. 3). Only 13 participants (12.9%) transmitted more than 500 GPS coordinates. Over 24 hours, the DCT app records approximately 500 GPS coordinates.

To quantify the total number of hours that could have been recorded via the DCT app by all participants during the study period, the *maximum Hours Recordable (*mHR*)* was calculated.

1 $$mHR=14\ days\ x\ 24\ hours\ x\ 141\ participants=\textrm{47,376}h$$

Equation 1. Calculation of maximum Hours Recordable (mHR).

**Fig. 4:**
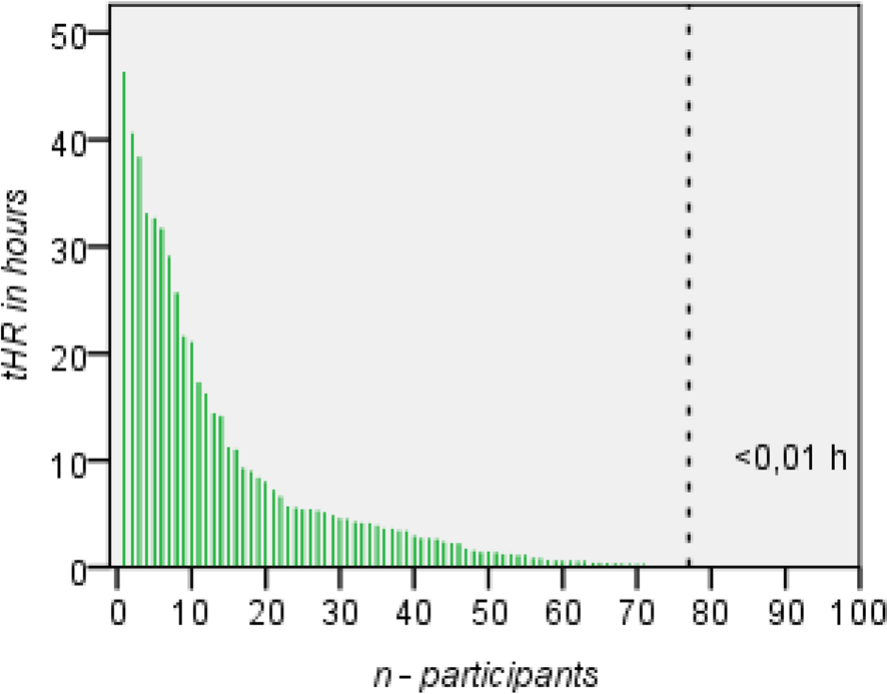
True Recorded Hours (tHR) in hours per participant over 14 days (total 469.3 h). * Poor adherence (GPS), battery consumption, energy supply, smartphone quality, technical experience

**Fig. 5:**
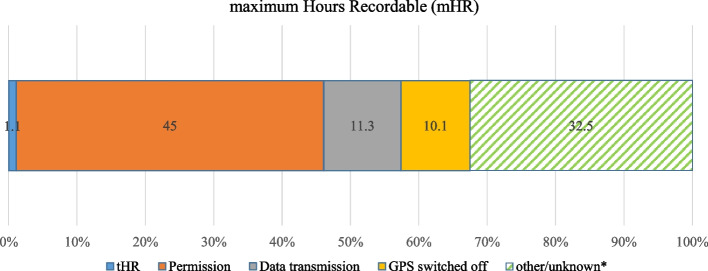
Maximum Hours Recordable (mHR) broken down into total hours recorded (tHR) and causes for non-tracking

The actual number of hours recorded by the participants during the study period (Fig. 4), *true Hours Recorded* (tHR), was 496.3 h. This tHR amounts to 1.1% of mHR, which explains the small number of GPS coordinates that were transmitted to the server. However, to understand the difference between mHR and tHR, we have to consider three main factors (permission, data transmission, GPS) and other observed factors, as displayed in Fig. 5.

During the first visit, it was established that in 127/141 (90.1%) smartphones, no GPS tracking was possible, as the needed permissions - ‘Location’, ‘Phone’ (Bluetooth, mobile data), ‘local storage’- were not automatically set. To remedy this issue, ‘manual permission adjustment’ was performed (Fig. 1). Over a duration of 7 days, 127 participants recorded no GPS coordinates, which means that 21.336 h of mHR (45.0%) was explained by a delayed permission set. Of the 14 participants (9.9%) for whom permissions were automatically set, 11/14 (78.6%) had Android 6 or earlier versions. The overall distribution of smartphones with Android 6 and earlier in the group of all participants was 21.5%. Newer Android versions (7 and later) are correlated **(***p* < 0.001) with blocking the DCT app from automatically using GPS/Location, Bluetooth and Memory.

Transmission failures of location data to the server have been identified as the second explanatory factor for the difference between tHR and mHR. Data transmission failures were associated with poor mobile reception or insufficient mobile data credit. The proportion of failed data transmissions from the 101 participants who sent GPS coordinates to the server during the visits is outlined in Table 2.

**Table 2:** Failed data transmission as a reason for reduced tHR and its proportion of mHR (11.3%, see Fig. 5);GPS and Bluetooth status during visits presented as % of participants

	*No Data transmission n (%)*	*Reason*	*Cumulative tHR h (% of mHR)*	*GPS on*	*Bluetooth on*
1st Visit	91/101 (*90.1%*)	87/101 (86.1*%*) permission not set 4/14 (*28.6%*) permission set but poor signal reception	384 (0.8%)	88.9	96.5
2nd Visit	42/101 (*41.6%*)	15/101 (*14.9%*) did not attend 27/101 (*26.7%*) insufficient data credit, poor signal reception	3024 (6.4%)	89.7	87.2
3rd Visit	16/101 (*15.8%*)	16/101 (*15.8%*) insufficient data credit, poor signal reception	1920 (4.1%)	91.0	82.4

During the first visit, data transmission to the server failed in 91/101 cases (90.1%), of which 87 were due to delayed manual permission adjustment. Out of 14 participants who had set automatic permissions successfully, 4/14 (28.6%) did not transmit data to the server. These four participants account for 384 h (0.8% of mHR) of missing GPS data logs due to failed data transmission over 4 days prior to the first visit. Out of the ten participants for whom data were successfully sent to the server, 70% were using previously tested smartphones, which is a higher proportion compared to 28.6% overall. During the second visit, data were not received by the server from 42/101 (41.6%) participants. Over 3 days (intervention until Day 7), these 42 participants accounted for 3024 h (6.4% of mHR) of missing GPS data logs. During the third visit, 16/101 (15.8%) participants could not transmit their data to the server. Over 5 days, 16 participants accounted for a further 1920 h (4.1% of mHR) of missing GPS data logs. The total number of hours for which no GPS coordinates were tracked due to transmission issues to the server is 5328 h (11.3% of mHR).

Additionally, it can be concluded that the significant (*p* < 0.001) reduction in data transmission failures between the first and third visits and between the second and third visits (Table 2) can be explained by the change in locations. The first two visits took place on the ground floor of a multistory building. In contrast, data transmission failures to the server were significantly reduced during the third visit, which took place in a single story building. The effect of location change may be underestimated, as the number of participants reporting insufficient mobile credit to transmit data increased throughout the process, peaking as an issue during the third visit.

During all visits, GPS and Bluetooth settings were checked. Identified as the third main factor to explain the difference between tHR and mHR, these results are presented in Table 3. Assessing all three visits, a mean of 10.1% participants had switched their GPS off, which corresponds to 4785 h (10.1% of mHR) during which no GPS coordinate logging was possible. This factor is likely to be underestimated because the analyzed data showed that participants switched on their GPS and Bluetooth settings directly before and switched it off after the visit. These results imply poor participant adherence to app use. However, a quantitive approximation of the real app run time cannot be estimated on the basis of tracked GPS coordinates due to multiple biases impacting the frequency of GPS coordinate logging.

**Table 3:** Relevant observations made concerning performance of DCT app

*Adherence to app use*	GPS was switched on prior and switched off after the visit
*Battery consumption*	Survey yields increased battery consumption, which likely reduced adherence
*Energy supply*	participants could not charge their Smartphone regularly due to limited access to electricity
*Smartphone deficiency*	corrupted memory cards; insufficient capacity of local storage; software errors (due to replicated Smartphones or Android OS, phone froze or had to be formatted)
*technical experience*	participants with little technical experience had to be provided with repeated training on how to use the DCT app correctly

Table 3 provides explanations for the remaining 32.5% of the difference between tHR and mHR that could not be quantified. One of the issues relates to battery consumption by the DCT app (see survey results in Additional File 4). Limited access to electricity during the study, coupled with higher app-related battery consumption, was a relevant limitation to the performance of a DCT app and may explain a reduced adherence to app use. Further differences between tHR and mHR may be attributed to deficiencies of participant smartphones with various reported software and hardware malfunctions (Table 3). The difference (*p* = 0.037) between the proportion of not previously tested brands in the overall group of participants (71.4%) and in the subgroup for which no GPS data were received by the server (90.9%) supports this assumption.

Some participants with limited technical know-how required repeated training on how to correctly use the DCT app. There was no significant correlation between tHR and participant profession or age.

### Effectiveness of aCT

**Fig. 6:**
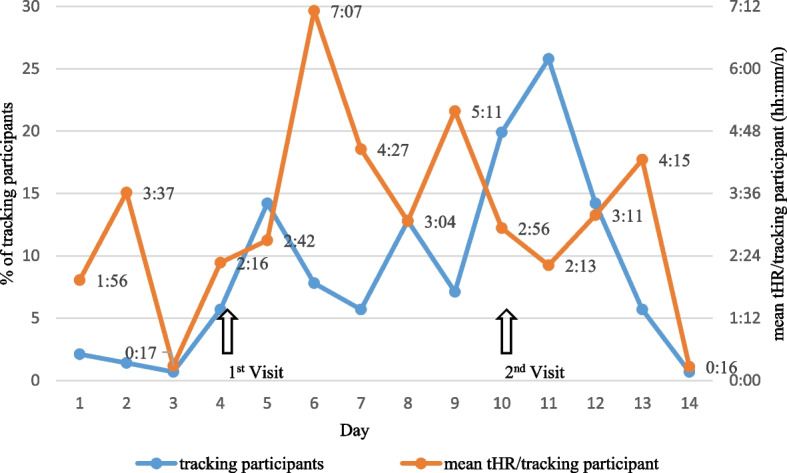
Percentage of participants logging GPS coordinates per day from study group of 141 participants; Mean time of tHR from participants logging GPS coordinates per day

To assess the DCT app effectiveness, we assembled aCT hits and pCT hits of all participants designated as infected, with corresponding matches (Table 4). To evaluate the extent of tracking activity, Fig. 6 displays the percentage of tracking participants and the mean tHR per day. By pCT, 1075 hits of designated infected participants with other participants have been listed, resulting in a median of 25.8 (± 28.8) hits per day per designated infected participant. A total of 147 pCT entries were not analyzed due to incomplete documentation. With aCT, five hits with a median of 0.1 (± 0.6) hits per day per designated infected participant were recorded. Of ten designated infected participants, only four were recording GPS coordinates during the infected period, of whom one participant only recorded one GPS coordinate. All designated infected participants recorded 4.3% (42.4 h tHR) of the time they were infected (984 h mHR) and a median of 62.1 minutes per day per infected person. The average time all participants logged GPS coordinates was significantly lower (*p* < 0.001) at 15.1 minutes per day per person.

**Table 4:**
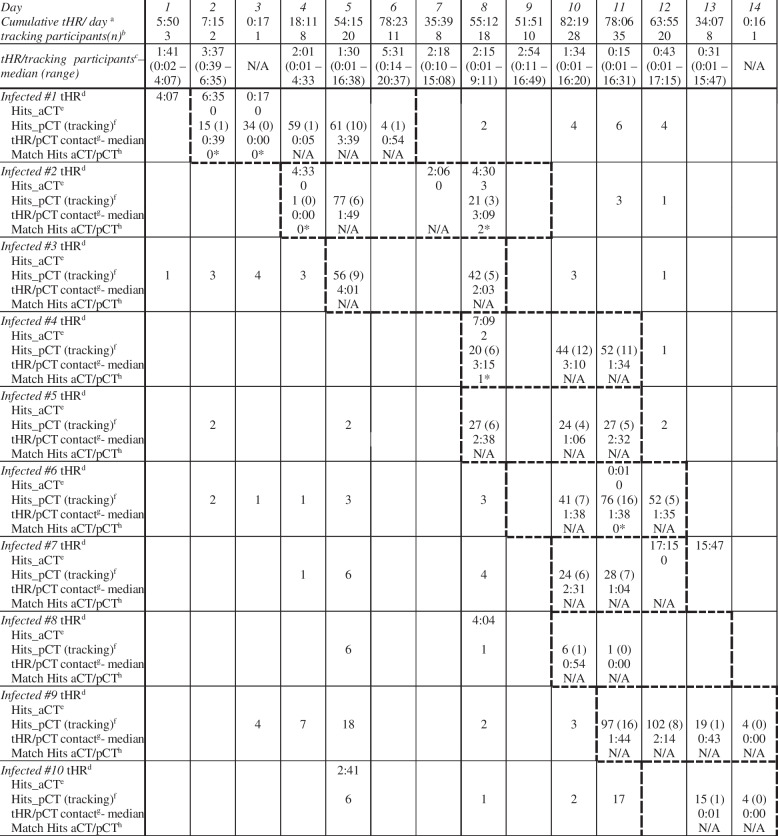
The number of tracking participants and the cumulative tHR per day such as the median of tracked hours of tracking participants was added, to assess the extent participants were tracking with the DCT app. For every *Infected* their tHR per day, their hits recorded with aCT and pCT with the number of those pCT contacts tracking on this day and their tHR on this day, together with the matches of aCT and pCT hits (further analyzed in Table 6) was outlined

^a^the sum of aCT tracked hours across all 141 participants on a given day (hh:mm)

^b^the number of tracking participants on a given day (n)

^c^the median of tracked hours of tracking participants on a given day (hh:mm)

^d^tracked hours of the participant designated as infected (1–10) on a given day (hh:mm)

^e^Hits of the participant designated as infected recorded via aCT (n)

^f^Hits of the participant designated as infected recorded via pCT and the number of those contacts tracking on this day (n)

^g^the median of tracked hours across all pCT contacts on a given day (hh:mm)

^h^the number of matches between aCT and pCT hits (n)

*Matches between recorded aCT hits and pCT hits (Match aCT/pCT) are further analyzed (Table 7)

This provides an explanation for why only five hits were recorded via the DCT app compared to the possible 1075 pCT listed hits. Of those five aCT hits, two were also recorded via pCT and could be accounted for as matches. Two aCT hits were recorded via Bluetooth pairing while GPS was inactive; thus, the DCT app used prior logged GPS coordinates that no longer reflected current location and resulted in a false-positive hit. One aCT hit was not listed via pCT by the designated infected participant but by the contact person and thus could be identified as a true aCT hit. In this case, the hit was recorded via aCT but not via pCT, demonstrating that the DCT app can be superior to a paper-based system on the precondition that both participants use the DCT app correctly.

Table 5 shows the contingency table evaluating the effectiveness of the DCT app. If every hit was a true hit, pCT found 1075/1077 (99.8%), and aCT found 5/1077 (0.5%). If only hits were analyzed when both participants had switched on their GPS, all three pCT hits were also recorded via aCT (plus one true aCT hit that was not listed via pCT). This suggests a high sensitivity of the DCT app under efficacy conditions. Determination of reliable thresholds for sensitivity or specificity was not possible due to the reduced time of tracked movement (tHR).

**Table 5:** Contingency table evaluating effectiveness of DCT app (aCT) versus paper based method (pCT) on the basis of recorded hits

	pCT positive	pCT negative	total
aCT positive	3	2	5
aCT negative	1072	0	1072
total	1075	2	1077

To determine if time stamp differences between GPS and Bluetooth pairing logged coordinates (periods where GPS was switched off, but Bluetooth was switched on, and thus Bluetooth pairing did not reflect the current location) were a relevant problem, the movement profiles concerning this matter were analyzed. Of the 101 participants where GPS coordinates were sent to the server, 47 participants (46.5%) showed periods of Bluetooth pairing and switched off GPS between one to ten times during the study, with a median time of 3:33 (0:31–14:09) h. Considering that 46.5% of the DCT app users for 3:33 h had an augmented possibility to record false-positive hits, this was identified as a significant impairment under real-world conditions.

To further examine the performance of the DCT app, all days on which designated infected participants tracked their movement using GPS with no aCT hits being recorded were analyzed (Table 6). There was no pCT hit where both infected and regular participants tracked their movement using GPS but no aCT hit was recorded. Consequently, no false negative results for aCT were found.

**Table 6:** Analysis of days participants designated as infected and according pCT contacts were tracking to check for false negative hits

	Day	Analysis
Infected #1	2	1 pCT contact was tracking on this day. Contact started tracking 6 h after pCT hit.
	3	0 pCT contacts were tracking on this day.
Infected #2	4	0 pCT contacts were tracking on this day.
	7	0 pCT contacts on this day.
	8	3 pCT contacts were tracking on this day. Of those 1 started tracking > 1 h after pCT hit and 2 were recorded via aCT (matches).
Infected #4	8	6 pCT contacts were tracking on this day. Of those 5 started tracking > 1 h after pCT hits and 1 was recorded via aCT (match).
Infected #6	11	16 pCT contacts were tracking on this day. Infected #6 tracked for one minute, which was > 1 h before or after pCT hits.
Infected #7	12	0 pCT contacts on this day. During 17:15 hours of tracking no aCT- hit was recorded. (see Additional file 5)

### Accuracy

**Fig. 7:**
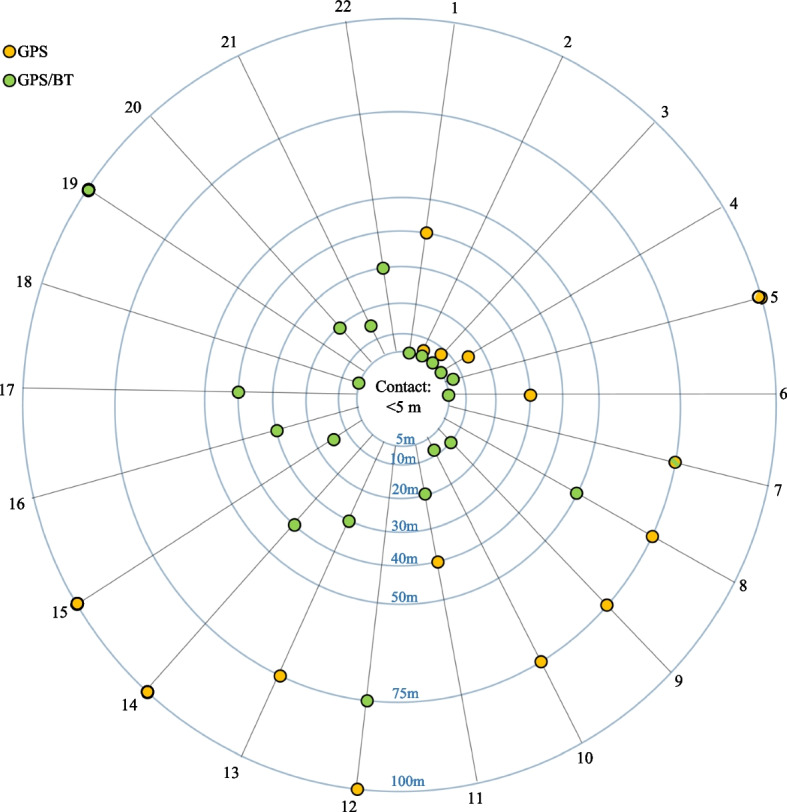
Accuracy in buildings. Contact of 22 participants with an index smartphone within a distance < 5 m in multi-story building. Green dots represent distances derived from both GPS and Bluetooth. Orange dots represent distance derived from GPS only

To evaluate aCT accuracy in buildings, an index smartphone was positioned next to participants. To detect whether Bluetooth increases accuracy in buildings as suggested, a comparison was made between aCT hits based on coordinates logged by both GPS and Bluetooth pairing and those based on GPS alone. For 7/22 participants, the hit only was recorded using additional coordinates logged by Bluetooth pairing. The results shown in Fig. 7 demonstrate that in buildings, coordinates logged by Bluetooth pairing increase the accuracy of the DCT app. The mean distance recorded by the DCT app using GPS and Bluetooth was 22.9 m ± 21.6 SD and was significantly (*p* = 0.004) more accurate than 60.9 m ± 34.7 SD recorded only via GPS coordinates. No significant correlation between recorded distance and phone brand or version of the smartphone OS was found.

**Fig. 8:**
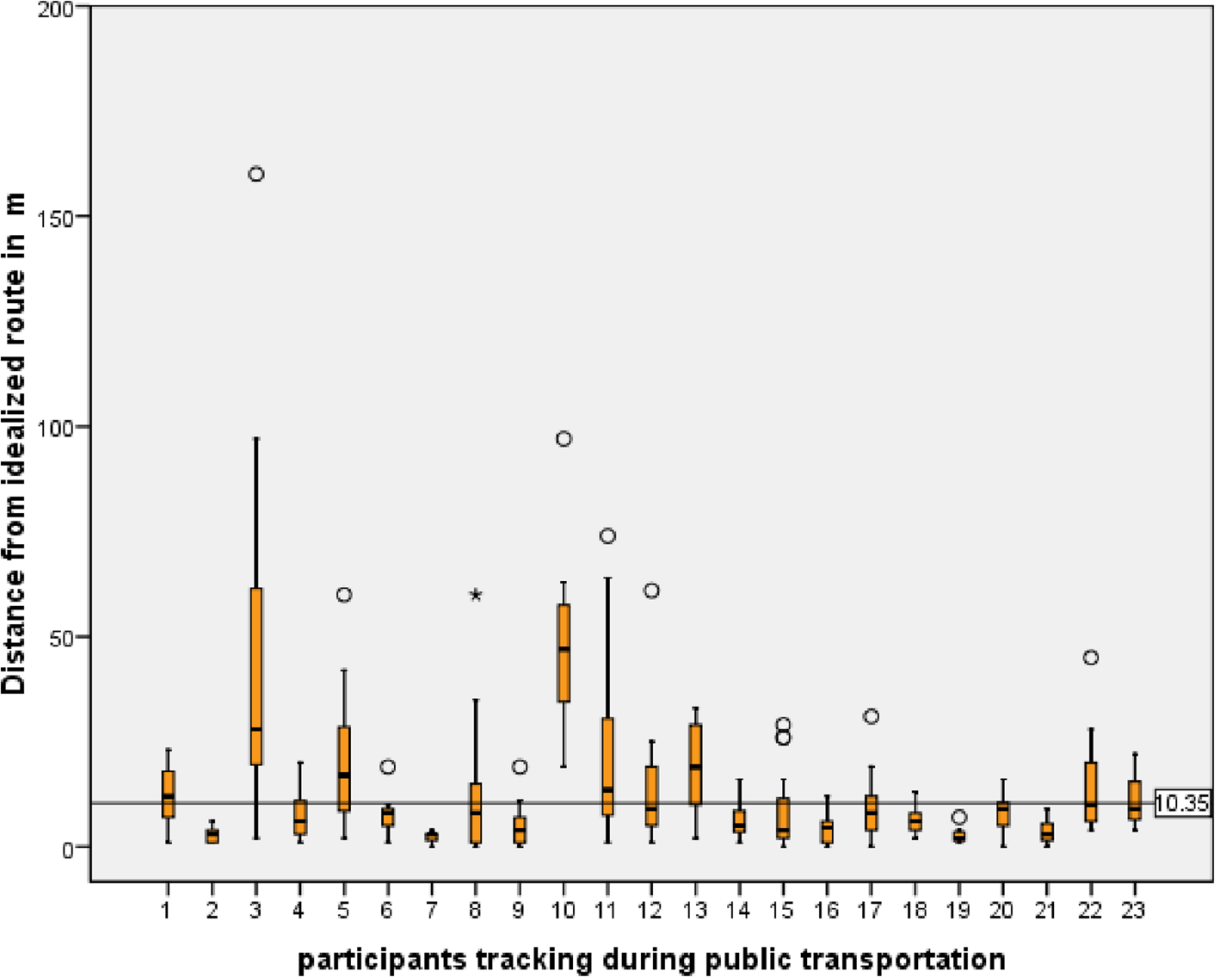
GPS accuracy in public transportation (mean 10.35 m); tracked population was 23 participants

To estimate accuracy in public transport, participants were asked to track their route to work using the DCT app. The precision of every GPS coordinate to the track was measured and illustrated in a boxplot (Fig. 8). The mean distance between the idealized route and recorded GPS coordinate as an approximation for GPS accuracy is 10.3 m ± 10.05 SD. There was a significant (*p* = 0.007) correlation between precision and phone brand, but no correlation with smartphone OS was found.

**Fig. 9:**
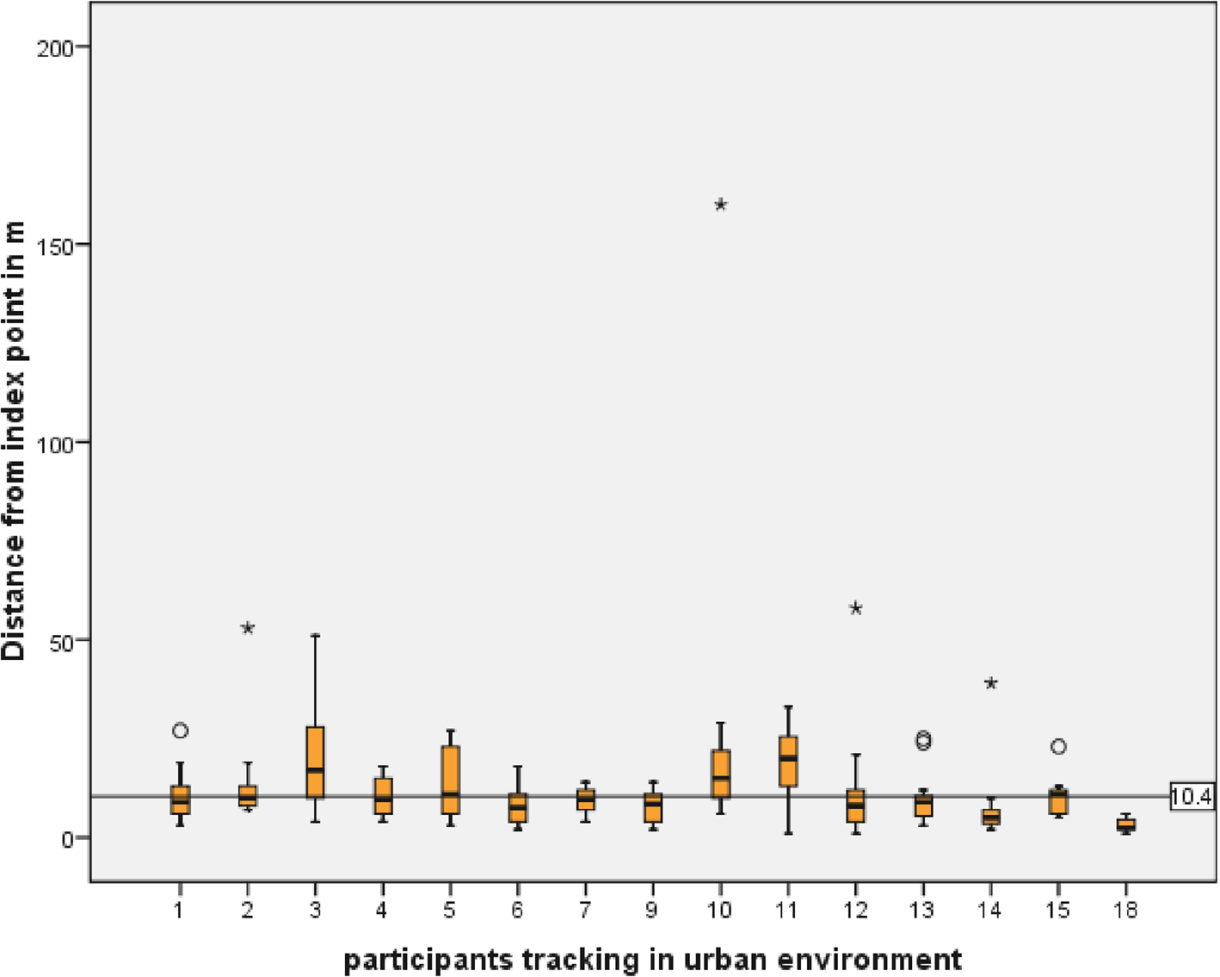
GPS accuracy outdoors in urban environment (mean 10.4 m); tracked population was 18 participants

To evaluate the accuracy outdoors in an urban environment, GPS coordinates of participants positioned at distinct locations were logged. The precision of logged GPS coordinates in relation to distinct location points was measured and illustrated in a boxplot (Fig. 9). The mean distance between the distinct location and recorded GPS coordinate as an approximation for GPS accuracy outdoors is 10.4 m ± 4.2 SD. No significant correlation between degree of precision and phone brand or the version of the smartphone OS was found.

### Identified operational factors influencing the feasibility of DCT

Additional findings that could be relevant for the implementation of a DCT app in low-income settings are as follows:

- The overall acceptance of the DCT app was high (see Additional File 6).
- The understanding about contact tracing in the study group (experience from the Ebola epidemic in 2014–2016) and the need for improvement in this field was good, which suggests a promising estimation for possible app uptake.
- To meet all specific demands and limit further burdens, close cooperation with local health officials and the Ministry of Health was identified as a main operational factor.
- When the DCT app was presented to ICT (information and communication technology) providers in Liberia, they confirmed that it would be possible to provide all mobile data from the DCT app free of charge to the user. The option for ICT providers to push the DCT app to its users for automatic installation and activation during a pandemic was confirmed.

## Discussion

Conclusions about the effectiveness of DCT are based on findings of studies conducted in countries with predominantly high- or middle-income settings. Additional research is necessary to identify specific burdens for the implementation of such techniques in low-income setting countries.

### Effectiveness of DCT

The effectiveness of mobile app-based DCT in real-world conditions of low-income settings has been identified as a major challenge by this study. High variability in smartphone quality, low levels of adherence to apps, insufficient access to mobile internet and unreliable access to electricity supply all inhibit DCT effectiveness. Many of these findings are likely to be an issue in other low-income regions as well and should be considered by further initiatives implementing mobile health tools. Sachs et al. [2] reported similar findings while introducing a mobile health tool during the 2014–16 Ebola epidemic in West Africa. There are recent studies on the effectiveness of DCT apps in real-world settings in La Gomera, Spain [11], Switzerland [12], Norway [13] and the UK [14] that conclude positively about the performance of the app. The results are difficult to compare with our findings because the effectiveness was estimated via indirect parameters due to the decentralized approach of the apps. A prospective study exploring the DCT app used in Australia [15] used a centralized approach and thus could compare DCT and MCT hits. The reported findings on effectiveness were similar to our results, and they concluded that the app did not make a meaningful contribution to COVID-19 contact tracing. Explanations for why many countries struggle to exploit the potential of DCT as predicted by modeling studies [3, 16–18] are the focus of current research. Based on a review of the literature [9], privacy concerns over user data, low trust in government and third parties (big data analysis, malicious actors), security vulnerabilities (hacker attacks), ethical issues (discrimination of minorities), user behavior and participation (limited experience with mobile devices, reasons for adoption) have been prioritized in primary studies, in contrast to understanding technical constraints. This is likely indicative of concerns around adoption of a DCT app dominating technical limitations in high income settings, which reflects the view of countries where primary studies have been conducted. According to our study results, technical constraints are a greater barrier to DCT implementation in low-income setting countries. Factors such as battery consumption in combination with limited energy supply, limited internet access (costs for mobile data, network coverage), hardware issues (smartphone quality, Bluetooth compatibility), and software issues (Android OS fragmentation, replicated software) should be the focus of further research.

### Different approaches of DCT

The studied DCT app is a location-based approach to DCT, using GPS to calculate proximity to infected individuals, while most initiatives of DCT have been based on proximity inference via Bluetooth Low Energy (BTLE). Using BTLE over GPS would reduce battery consumption, partly mitigating unreliable energy supply effects on DCT effectiveness. Technical reliability limitations of BTLE have been reported [19, 20]. Our finding that newer versions of OS impede proper app use has been reported by other initiatives and was addressed by the new Apple/Google Application Programming Interface (API) [21]. However, BTLE is an emerging technique and cannot be applied on every smartphone, especially in low-income settings. Given that more than 40% (one billion) of Android active users worldwide use version 6.0 or below and no longer receive updates, many Android devices may not benefit from updates to the new BTLE-based contact tracing system Google built in collaboration with Apple during the COVID-19 pandemic (Google/Apple API) [10]. High app uptake rates have been identified as a crucial factor in DCT effectiveness [3, 17, 18], and unsupported OS versions could prevent achieving the required uptake rates in low-income settings if BTLE is used. Of the participants in our study (urban population, hospital staff), 20% were using Android 6 or below, whereas in the overall population, the number could be significantly higher. Thus, a large proportion of smartphone owners in low-income settings would be excluded from DCT if BTLE techniques are selected. Additionally, the existing difference in access to health care due to DCT for disadvantaged communities caused by limited availability of smartphones and internet access [22, 23] would be further exacerbated by implementing a DCT app requiring BTLE. In contrast, the location-based Bluetooth-assisted GPS method used in this study mitigates those barriers, as it utilizes widely available technology.

To address the problem that data transmission of the DCT app was often limited due to insufficient mobile data credit, we strived for a solution to provide mobile data for the DCT app free of charge. Approached ICT providers were willing and able to realize that in the event of an epidemic. Affordability of mobile internet is a burden to digital inclusion in disadvantaged communities, especially considering that the median cost of 1 GB of mobile data is 15.3% of monthly GDP per capita for the poorest income quintile [24]. We recommend that other initiatives seeking to implement DCT in low-income settings provide DCT free of charge to end users. Including ICT providers in the deployment of a DCT app is also crucial to ensure sufficient network coverage and, if applicable, remote installation of the app.

Other GPS-based DCT apps have been used in South Korea (Corona 100), China, Israel (Ha’Magens) or the USA (Private Kit) with varying success [9]. To date, few peer-reviewed papers on the effectiveness of these apps have been published. The impairment of GPS accuracy in urban settings, as seen in our results, is consistent with other studies [25, 26]. Although the Bluetooth-assisted GPS location-based DCT app improved accuracy in buildings, this came at the cost of a higher risk of recording false-positive hits when GPS was switched off. To reduce this risk, the interval between the timestamp of the last recorded GPS coordinate and the timestamp of coordinates based on Bluetooth pairing needs to be matched. High numbers of false-positive exposure notifications raise doubts about the usefulness of the app in the population, as reported for the Ha’Magens app in Israel [27], a location-based DCT app used as an automated tool for DCT.

Impairment of GPS accuracy in urban settings together with the other DCT limitations identified suggests using DCT to supplement traditional manual contact tracing. In case of an infection, the app users can voluntarily look at a map with their logged GPS coordinates, which can only be accessed together with a verified health official (e.g., contact tracers). The ‘recall problem’ has often been described as one of the weaknesses of manual contact tracing [28]. Listing the possible contacts during a period of time, e.g., the last 2 weeks, from memory alone is often insufficient. By using GPS coordinate logging of the last 2 weeks, prospective contact tracing is likely to be more accurate and effective. Another strategy that was reported as highly successful during the COVID-19 pandemic, but rarely implemented, is retrospective cluster-based tracing [29]. The intention is to recognize infection clusters early by asking where people have been infected and thereby informing other people from the cluster who might have been infected but who are still presymptomatic or asymptomatic. One reason retrospective cluster-based tracing was not commonly used is that the additional tracing effort might be beyond the capacity of public health officials during the surge of a pandemic. Aggregated GPS coordinate logs of infected persons are able to reveal clusters of infection early and thus enable containment measures to be implemented sooner.

### Drivers and barriers of DCT

High trust in authorities and health officials is an important factor in achieving high rates of app uptake, acceptance and adherence [27, 30, 31]. Misinformation and poor transparency surrounding data privacy have led to some DCT attempts being unsuccessful [27]. South Korea applied DCT requiring high levels of sensitive data access (geolocation, medical records, camera, financial transaction), yet due to transparent and accurate information communicated by the government, the population demonstrated trust in the app, which boosted the effectiveness of contact tracing. When surveyed, 86% of the population stated that the government had done ‘a good job’ dealing with the pandemic and that their country was more united as a result [32]. In contrast to many East Asian societies, there is little evidence that this level of privacy intrusion could be replicated within European societies due to their historical skepticism toward state surveillance [7, 9, 33, 34]. This highlights that social groups might differ in their judgment of fundamental considerations based on cultural differences, social preferences or individual risks. Deliberation of privacy concerns and DCT effectiveness (voluntary vs. mandatory, decentralized vs. centralized approaches) should be considered within the specific context of epidemics. Given epidemics with high case fatality rates, high numbers of infected individuals [35] and a collapsing health system (e.g., the Ebola epidemic of 2014–2016), it is likely that measures for effective disease containment could be valued higher than data privacy concerns by the population. Perceived personal threat and lack of personal control are positively related to the acceptance of surveillance technologies [36, 37]. This is consistent with our study results, where a high overall acceptance of a DCT app was identified in a cohort of health workers who had previously also been confronted with a humanitarian crisis during the Ebola epidemic in 2014–16. In spite of this, however, we still detected a discrepancy between high reported overall acceptance of DCT and low levels of adherence to app use during the study. The high rates of switched off GPS, seen in the results of this study, in large parts can be explained by increased battery consumption in combination with limited energy supply (some participants could not use their power generator due to shortage of fuel during the study). Resolving the burden of limited internet access would increase the number of hours the DCT app tracked by 11.3%. Additional factors such as smartphone quality and technical experience have been identified to explain reduced compliance.

The detected reasons for the finding that only 1.1% of the maximum Hours Recordable (mHR) during the study period was recorded by the participants using the DCT app (tHR) are consistent with the known drivers and barriers to uptake of DCT, which can be classified into four categories: motivation, access, skills and trust [38]. The main kinds of motivation are changes in the perception of risk and perception of effectiveness of DCT, convenience of app use (e.g., battery consumption, mobile data), the bandwagon effect and whether DCT is mandatory or voluntary, which have a huge influence on app uptake. The accessibility (affordability/availability) of the necessary equipment for DCT (internet, electricity, hardware and software) determines whether people can participate in DCT. A sufficient level of technical or literacy skill and an easy-to-understand format of information are required to understand what DCT is and how it works. Trust in entities associated with DCT, such as governments, corporations (Google, Apple, etc.), data architecture (centralized vs. decentralized) and data security biases the decision of people to use DCT.

The decision to participate in DCT or not depends on the complex interaction of these factors. A dependency on individual control of many of these factors has been reported [38]. Because during an epidemic the sense of loss of control is likely to increase, an essential question with respect to people’s decisions to participate in DCT is: do people believe that their choices and actions create a positive change? Large-scale surveys [39] found that individual control is correlated with perceived knowledge. Giving people information about (effective) DCT influences their sense of control and can lead to high levels of uptake and compliance because of the feeling of contributing to containing the epidemic.

To ensure effective communication at the community level and to reach vulnerable and disadvantaged groups, we implemented a feature in the DCT app where relevant information could be shared:

- Education on the disease (written and illustrative images)
- Working principle, benefits and limitations of the DCT app
- Updated information on the outbreak and corresponding measures
- Addressing of public misinformation

Managing misinformation is an important component of a response strategy because, as seen during the Ebola epidemic in West Africa in 2014–16 [1, 39] and the COVID-19 pandemic [40], misinformation can inhibit effective epidemic containment. To meet the functional criteria and minimize the bureaucratic burdens for the implementation of a DCT app, we identified the involvement of health authorities (e.g., MoH) as an important operational success factor. Management and maintenance of a DCT app requires a considerable amount of qualified personnel, skills and experience from local health officials and institutions. Additionally, proactive engagement of community leaders and religious or cultural figures should be encouraged for high rates of acceptance throughout the population [38].

## Conclusion

DCT is feasible as a supplement to traditional manual contact tracing. The finding that only 1.1% of the maximum Hours Recordable (mHR) during the study period was recorded by the participants using the DCT app (tHR), several limitations of the DCT found in our study together with the impairment of GPS accuracy in urban settings impede the sole use of a DCT app. The substantial additional workload of managing a DCT app calls for supportive evidence of high rates of effectiveness to justify the significant investment of a DCT app by low-income countries.

## Limitations

To conduct this study, a Beta version of the DCT app was used to identify potential improvements prior to Version 1.0. The beta version was engineered only for Android OS.

The selected study population is not representative of the overall population in low-income settings, due to the biases of participants all being employed and thus being economically more stable than the average population.

Although the paper-based approach enables only low precision measurements of distance and time, it provided the most accessible option to ensure documentation of contacts between participants as a reference standard for the DCT app.

The focus of this study was on the feasibility of DCT; consequently, little data about the functionality of the used smartphones was collected. In a further study, data about the smartphone operating time, e.g., exact time of switching on GPS/Bluetooth, power saving modes, runtime of the DCT app and the smartphone, usage of other apps and interfaces or devices in the environment that interfere with the smartphone, would help to further develop a DCT app for the targeted population.

## Supplementary Information

**Additional file 1.** Software code - This document contains the java script based software code to analyze contacts between participants.

**Additional file 2.** Accuracy public transportation - Measurement of accuracy during use of public transportation.

**Additional file 3.** Smartphone models – Detailed information of used smartphones with number of logged GPS coordinates.

**Additional file 4.** Battery consumption - Survey results on battery consumption.

**Additional file 5.** Infectet # 7 - Logged GPS coordinates (yellow tags) of Infected #7 on day 12 were analyzed to check for possible false negative results. There were no aCT hits despite 17:15 hours tracking, due to the fact that Infected #7 was in the hospital only at nighttime and large parts of the tracked time was spend remote from the hospital, where he did not meet other participants. Furthermore a big variance of GPS locations is displayed.

**Additional file 6.** Overall acceptance – Survey results on overall acceptance of the DCT.

## Data Availability

The datasets generated and analysed during the current study are not publicly available due to legal guidelines for data security and compromised individual privacy.

## Abbreviations

aCT: App-based contact tracing
API: Application Programming Interface
BT: Bluetooth
BTLE: Bluetooth Low Energy
COVID-19: Coronavirus Disease 2019
DCT: Digital Contact Tracing
Fig.: Figure
GB: Gigabyte
GDP: Gross domestic product
GPS: Global Positioning Service
h: Hours
ICT: Information and Communications Technologies
IQR: Interquartile range
JFK: John Fitzgerald Kennedy
m: Meter
MCT: Manual Contact Tracing
mHR: Maximum Hours recordable
MoH: Ministry of Health
OS: Operating System
pCT: Paper-based contact tracing (reference system)
SD: Standard Deviation
tHR: true Hours Recorded
WHO: World Health Organization
