# Supplementary material for: Feasibility of digital contact tracing in low-income settings – pilot trial for a location-based DCT app
Source: BMC Public Health. 2023 Jan 21;23:146. doi: 10.1186/s12889-022-14888-x (PMC9859743; doi:10.1186/s12889-022-14888-x)
Supplement: Supplementary file 1 — Additional file 1. Software code - This document contains the java script based software code to analyze contacts between participants. [file 12889_2022_14888_MOESM1_ESM.pdf]

Feasibility of digital contact tracing in low-income settings – pilot trial for a location-based DCT app

Journal: BMC Public Health

Eric Handmann, MD (first author, corresponding author)
Department for Emergency Medicine, University Hospital Leipzig, Leipzig, Germany
Mail: Eric.Handmann@medizin.uni-leipzig.de; ORCID #0000-0001-7584-007X
Sia Wata Camanor, Mosoka P. Fallah, Neima Candy, Davidetta Parker, André Gries, Thomas Grünewald

AF 1 This document contains the java script based software code to analyze contacts between participants

```
// C4:93:D9:27:18:71
//SELECT Cases_Region,Cases_Data FROM `Cases` WHERE DATE(Cases_Date) >=
'2020-02-01' AND length(Cases_Data) > 2 GROUP BY Cases_Region ORDER BY
Cases_Region;

var selector1 = document.getElementById('select_case1');
var selector2 = document.getElementById('select_case2');
var inputRange = document.getElementById('inputRange');
var inputTime = document.getElementById('inputTime');
var submitButton = document.getElementById('submit_button');
var output = document.getElementById('output');
var selectedCase1 = {};
var selectedCase2 = {};
var cleanedCases = cleanCases();
var mergedDuplicates = mergeDuplicates();
//printAll();
var finalCases = removeDuplicateTimestamps ();
buildDropDown();
var foundCounter = 0;
var maxRange = 5;
var minTimePref = 2*60*1000;

selector1.addEventListener("change", function(){
    var i = selector1.options[selector1.selectedIndex].value;
    selectedCase1 = mergedDuplicates[i];
})

selector2.addEventListener("change", function(){
    var i = selector2.options[selector2.selectedIndex].value;
    selectedCase2 = mergedDuplicates[i];
})

inputRange.addEventListener("change", function(){
    maxRange = parseInt(inputRange.value)
})

inputTime.addEventListener("change", function(){
    minTimePref = parseInt(inputTime.value)
    minTimePref = minTimePref*60*1000;
})

submitButton.addEventListener("click", iterateCases);

function buildDropDown() {
    mergedDuplicates.forEach(function(obj, i){
        var option = document.createElement("option");
        option.text = obj.id+" ("+obj.data.length+")";
```

Eric Handmann, MD (first author, corresponding author)
Department for Emergency Medicine, University Hospital Leipzig, Leipzig, Germany
Mail: Eric.Handmann@medizin.uni-leipzig.de; ORCID #0000-0001-7584-007X
Sia Wata Camanor, Mosoka P. Fallah, Neima Candy, Davidetta Parker, André Gries, Thomas Grünewald

```
        option.value = i;
        selector1.appendChild(option);
        selector2.appendChild(option.cloneNode(true));
    })

}

function cleanCases() {
    var cleanedCases = [];
    cases.forEach(function(e,i){
        var obj = {};
        obj.id = e.Cases_Region;
        var tmpData = JSON.parse(e.Cases_Data);
        var resData = [];
        tmpData.forEach(function(ee){
            var innerObj = {};
            innerObj.timestamp = ee[ee.length-1];
            innerObj.lat = ee[0];
            innerObj.lng = ee[1];
                 innerObj.bt = ee[3];
            resData.push(innerObj);
        })
        obj.data= resData;
        cleanedCases.push(obj);
    });
    return cleanedCases;
};

function printAll() {
    cleanedCases.forEach(function(cs){
        cs.data.forEach(function(d){
            var myDate= new Date(d.timestamp);
            var dateString = myDate.getDate();
            if(dateString <=9) dateString = '0'+dateString;
            var hourString = myDate.getHours();
            if(hourString <=9) hourString = '0'+hourString;
            var minuteString = myDate.getMinutes();
            if(minuteString <=9) minuteString = '0'+minuteString;
            if(d.bt.length > 3)
            {
                bts = JSON.parse(d.bt);
                bts.forEach(function(bt){
                    var p = ''
                    p = cs.id+";"+d.timestamp+";"+myDate.getFullYear()+"-
0"+(myDate.getMonth()+1)+"-
"+dateString+";"+hourString+":"+minuteString+";"+d.lat+";"+d.lng+";"+bt+"
\\n";
                    output.append(p);
                })
            }
            else {
                var p ='';
```

Eric Handmann, MD (first author, corresponding author)
Department for Emergency Medicine, University Hospital Leipzig, Leipzig, Germany
Mail: Eric.Handmann@medizin.uni-leipzig.de; ORCID #0000-0001-7584-007X
Sia Wata Camanor, Mosoka P. Fallah, Neima Candy, Davidetta Parker, André Gries, Thomas Grünewald

```
                p = cs.id+";"+d.timestamp+";"+myDate.getFullYear()+"-
0"+(myDate.getMonth()+1)+"-
"+dateString+";"+hourString+":"+minuteString+";"+d.lat+";"+d.lng+";"+d.bt
+"\\n";
                output.append(p);
            }

        })

    })
}

function mergeIterator(current, previous) {
    var mergedResultData = [];
    previous.data.forEach(function(e){
        var found = false;
        tmp = e;
        var tmp2 = {}
        current.data.forEach(function(ee){
            tmp2 = ee;
            if(ee.timestamp == e.timestamp){
                found = true;
            }
        })
        if(!found)
            mergedResultData.push(tmp2);
        mergedResultData.push(e);
    })
    return mergedResultData;
}

function mergeDuplicates() {
    var result = [];
    var previousCase = cleanedCases[0];
    cleanedCases.forEach(function(e, i){
        if(i==0){
            result.push(e);
        }
        else{
            if(e.id == previousCase.id){
                e.data = mergeIterator(e,previousCase);
            }
            else result.push(e);
            previousCase = e;
        }
    });
    return result;

};
function removeDuplicateTimestamps(){
    mergedDuplicates.forEach(function(e){
        previousTimestamp = 0;
```

Eric Handmann, MD (first author, corresponding author)
Department for Emergency Medicine, University Hospital Leipzig, Leipzig, Germany
Mail: Eric.Handmann@medizin.uni-leipzig.de; ORCID #0000-0001-7584-007X
Sia Wata Camanor, Mosoka P. Fallah, Neima Candy, Davidetta Parker, André Gries, Thomas Grünewald

```
        tmpData = [];
        e.data.forEach(function(ee){
            if(ee.timestamp != previousTimestamp)
                tmpData.push(ee)
            previousTimestamp = ee.timestamp;
        })
        e.data = tmpData;
    })
    return mergedDuplicates;
}
function iterateCases() {
    output.innerHTML = "";
    var foundMatches = [];
    finalCases.forEach(function(e){
        selectedCase2 = e;
        //console.log(e.id);
        if(selectedCase1.id != selectedCase2.id){
            var c = evaluateCase();
            if(c.timestamps.length > 0)
                foundMatches.push(c);
        }
    })
    // now we have the timestamps for matches. Calculate time together

    foundMatches.forEach(function(e){
        timeSum = 0;
        e.timestamps = e.timestamps.sort();
        var previous = e.timestamps[0];
        var contactDates = [];
        var contactDuration = 0;
        var contactDurations = [];
        contactDates.push(new Date(previous));
        e.timestamps.forEach(function(ee){
        if(ee-previous < 900000 && previous != 0){
            timeSum += ee-previous;
            contactDuration += ee-previous;
        }
        else {
            contactDates.push(new Date(ee));
            contactDurations.push(contactDuration);
            contactDuration = 0;
        }
        previous = ee;
        })
        contactDurations.push(contactDuration);

        timeSum2 = 0;
        e.timestamps2 = e.timestamps2.sort();
        var previous2 = e.timestamps2[0];
        var contactDates2 = [];
        var contactDuration2 = 0;
        var contactDurations2 = [];
```

Eric Handmann, MD (first author, corresponding author)
Department for Emergency Medicine, University Hospital Leipzig, Leipzig, Germany
Mail: Eric.Handmann@medizin.uni-leipzig.de; ORCID #0000-0001-7584-007X
Sia Wata Camanor, Mosoka P. Fallah, Neima Candy, Davidetta Parker, André Gries, Thomas Grünewald

```
        contactDates2.push(new Date(previous2));
        e.timestamps2.forEach(function(ee){
            if(ee-previous2 < 900000 && previous2 != 0){
                timeSum2 += ee-previous2;
                contactDuration2 += ee-previous2;
            }
            else{
                contactDates2.push(new Date(ee));
                contactDurations2.push(contactDuration2);
                contactDuration2 = 0;
            }
            previous2 = ee;
            })
            contactDurations2.push(contactDuration2);

            if(timeSum > minTimePref || timeSum2 >minTimePref ) {

                var minTime = 0;
                var maxTime = 0;
                var refContactDates = contactDates;
                var refContactDurations = contactDurations;
                if(timeSum >= timeSum2){
                    minTime = Math.round(timeSum2/1000/60);
                    maxTime = Math.round(timeSum/1000/60);
                }
                else{
                    minTime = Math.round(timeSum/1000/60);
                    maxTime = Math.round(timeSum2/1000/60);
                    refContactDates = contactDates2;
                    refContactDurations = contactDurations2;
                }
                var p = document.createElement("p");
                p.innerHTML = "ID: "+e.id+", Kontaktzeit zwischen 
"+minTime + " und "+maxTime+" Minuten";
                output.appendChild(p);
                refContactDates.forEach(function(e,i){
                    var pp = document.createElement("p");
                    pp.innerHTML = "     -"+e+" 
Dauer: "+Math.round(refContactDurations[i]/1000/60)+" Minuten";
                    output.appendChild(pp);
                })
                var sel = document.createElement("select");
                output.appendChild(sel);
                tmpBT = []
                e.foundBT.forEach(function(bt,i){
                    if(bt.length>0) {
                        bt.forEach(function(b){
                            tmpBT.push(b);
                        })

                    }
```

Eric Handmann, MD (first author, corresponding author)
Department for Emergency Medicine, University Hospital Leipzig, Leipzig, Germany
Mail: Eric.Handmann@medizin.uni-leipzig.de; ORCID #0000-0001-7584-007X
Sia Wata Camanor, Mosoka P. Fallah, Neima Candy, Davidetta Parker, André Gries, Thomas Grünewald

```
                })
                tmpBT.sort();
                var counts = tmpBT.reduce((acc, value) => ({
                    ...acc,
                    [value]: (acc[value] || 0) + 1
                 }), {});
                var k = Object.keys(counts)
                k.forEach(function(ee,i){
                    var option = document.createElement("option");
                    option.text = ee+" ("+counts[ee]+")";
                    option.value = i;
                    sel.appendChild(option);
                })

        }
        return timeSum;
    })

    var div = document.createElement("div");
    div.innerHTML = "fertig";
    output.appendChild(div);
}

function evaluateCase() {
    foundCounter = 0;
    var foundMatches = [];
    foundMatches.timestamps = [];
    foundMatches.timestamps2 = [];
    foundMatches.foundBT = [];
    selectedCase1.data.forEach(function(data1){
        var previousTimestamp = 0;
        var previousTimestamp2 = 0;
        selectedCase2.data.forEach(function(data2){
            var min = data2.timestamp-(1000*60*1);
            var max = data2.timestamp+(1000*60*1);
            if(data1.timestamp >= min && data1.timestamp <= max) { //both
persons have entries at about the same time
                if(data1.bt.length > 1)
                        foundMatches.foundBT.push(JSON.parse(data1.bt));
                d =
distance(data1.lat,data1.lng,data2.lat,data2.lng,'K');
                if(d <= maxRange){
                    foundMatches.id = selectedCase2.id;
                    if(data1.timestamp != previousTimestamp)
                        foundMatches.timestamps.push(data1.timestamp);
                    if(data2.timestamp != previousTimestamp2)
                        foundMatches.timestamps2.push(data2.timestamp);


                    previousTimestamp = data1.timestamp;
                    previousTimestamp2 = data2.timestamp;
```

Eric Handmann, MD (first author, corresponding author)
Department for Emergency Medicine, University Hospital Leipzig, Leipzig, Germany
Mail: Eric.Handmann@medizin.uni-leipzig.de; ORCID #0000-0001-7584-007X
Sia Wata Camanor, Mosoka P. Fallah, Neima Candy, Davidetta Parker, André Gries, Thomas Grünewald

```
                    }

               }
          })
     })
     return foundMatches;
}

function distance(lat1, lon1, lat2, lon2, unit) {
      if ((lat1 == lat2) && (lon1 == lon2)) {
            return 0;
      }
      else {
            var radlat1 = Math.PI * lat1/180;
            var radlat2 = Math.PI * lat2/180;
            var theta = lon1-lon2;
            var radtheta = Math.PI * theta/180;
            var dist = Math.sin(radlat1) * Math.sin(radlat2) + 
Math.cos(radlat1) * Math.cos(radlat2) * Math.cos(radtheta);
            if (dist > 1) {
                  dist = 1;
            }
            dist = Math.acos(dist);
            dist = dist * 180/Math.PI;
            dist = dist * 60 * 1.1515;
            if (unit=="K") { dist = dist * 1.609344 }
            if (unit=="N") { dist = dist * 0.8684 }
            return dist;
      }
}
```
