# Supplementary material for: Feasibility of digital contact tracing in low-income settings – pilot trial for a location-based DCT app
Source: BMC Public Health. 2023 Jan 21;23:146. doi: 10.1186/s12889-022-14888-x (PMC9859743; doi:10.1186/s12889-022-14888-x)
Supplement: Supplementary file 2 — Additional file 2. Accuracy public transportation - Measurement of accuracy during use of public transportation. [file 12889_2022_14888_MOESM2_ESM.pdf]

**Feasibility of digital contact tracing in low-income settings – pilot trial for a location-based DCT app**

Journal: BMC Public Health

*Eric Handmann*, MD (first author, corresponding author)

Department for Emergency Medicine, University Hospital Leipzig, Leipzig, Germany

Mail: Eric.Handmann@medizin.uni-leipzig.de; ORCID #0000-0001-7584-007X

*Sia Wata Camanor, Mosoka P. Fallah, Neima Candy, Davidetta Parker, André Gries, Thomas Grünewald*

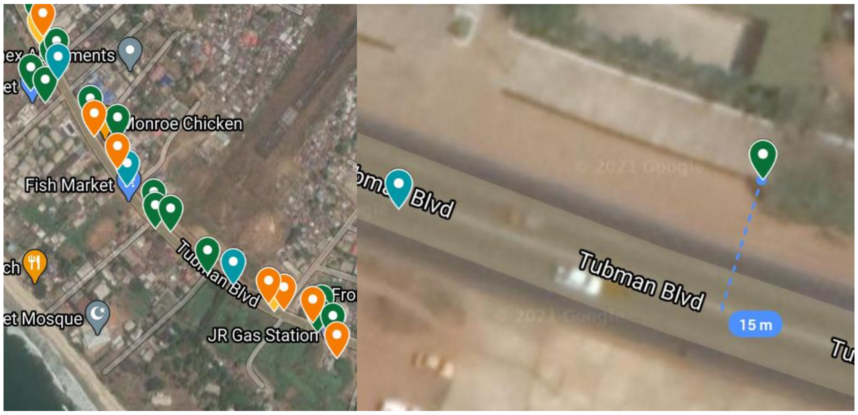


**AF 2** Measurement of accuracy during use of public transportation
