# Supplementary material for: Feasibility of digital contact tracing in low-income settings – pilot trial for a location-based DCT app
Source: BMC Public Health. 2023 Jan 21;23:146. doi: 10.1186/s12889-022-14888-x (PMC9859743; doi:10.1186/s12889-022-14888-x)
Supplement: Supplementary file 3 — Additional file 3. Smartphone models – Detailed information of used smartphones with number of logged GPS coordinates. [file 12889_2022_14888_MOESM3_ESM.pdf]

Feasibility of digital contact tracing in low-income settings – pilot trial for a location-based DCT app

Journal: BMC Public Health

Eric Handmann, MD (first author, corresponding author)

Department for Emergency Medicine, University Hospital Leipzig, Leipzig, Germany

Mail: Eric.Handmann@medizin.uni-leipzig.de; ORCID #0000-0001-7584-007X

Sia Wata Camanor, Mosoka P. Fallah, Neima Candy, Davidetta Parker, André Gries, Thomas Grünewald

AF 3: Detailed information of used smartphone models with number of logged GPS coordinates

Phone brand	Model	Android Version	Number of logged GPS coordinates
Tecno	cf7k	unknown	80
Tecno	spark pro	unknown	750
Tecno	Spark 2	unknown	0
Tecno	la7	unknown	2
Tecno	y2	4	0
Tecno	c8	5	17
Tecno	one 5	5	0
Tecno	j8	5	489
Tecno	W5	6	465
Tecno	l8 lite	6	1023
Tecno	L9Plus	7	368
Tecno	K7	7	2
Tecno	k8	7	251
Tecno	lc6	7	4
Tecno	Phantom 6 Plus	7	133
Tecno	l9 plus	7	0
Tecno	wx3p	7	0
Tecno	cx	7	306
Tecno	k7	7	13
Tecno	wx3	7	82
Tecno	f2	7	0
Tecno	camon cx air	7	1
Tecno	la7 pro	8	90
Tecno	LB6	8	64
Tecno	cf7k	8	53
Tecno	la7 pro	8	0
Tecno	cf8	8	0
Tecno	b1	8	0
Tecno	f1	8	0
Tecno	ca8	8	66
Tecno	ba2	8	1184
Tecno	ca8	8	3
Tecno	L c8	8	16
Tecno	ba2	8	45
Tecno	la7	8	5
Tecno	camon 11 cf7	8	2
Tecno	f1	8	0
Tecno	f1	8	194
Tecno	cf7k	8	1

Feasibility of digital contact tracing in low-income settings – pilot trial for a location-based DCT app
Journal: BMC Public Health
Eric Handmann, MD (first author, corresponding author)
Department for Emergency Medicine, University Hospital Leipzig, Leipzig, Germany
Mail: Eric.Handmann@medizin.uni-leipzig.de; ORCID #0000-0001-7584-007X
Sia Wata Camanor, Mosoka P. Fallah, Neima Candy, Davidetta Parker, André Gries, Thomas Grünewald

Tecno	Lb7	8	914
Tecno	lb6	8	119
Tecno	cf8	8	2
Tecno	ka7	8	0
Tecno	cf7	8	251
Tecno	kb7j	9	133
Tecno	cc6	9	5
Tecno	a6	9	187
Tecno	kb8	9	0
Tecno	cc7	9	3
Tecno	ab7	9	604
Tecno	lc6	9	0
Tecno	cc7	9	0
Tecno	KC8	9	0
Tecno	kc8	9	43
Tecno	kb7j	9	122
Tecno	cc7	9	1684
Infinix	zero	unknown	48
Infinix	hot 4	6	2005
Infinix	x608	8	246
Infinix	x5515	8	193
Infinix	x609	8	2
Infinix	X604	8	0
Infinix	unknown	9	0
Infinix	note 5	9	211
Infinix	x652	9	1
Itel	unknown	unknown	0
Itel	it1556 Plus	5	0
Itel	se	6	0
Itel	s31	6	0
Itel	s11	6	0
Itel	s32	6	0
Itel	s32	7	96
Itel	s32	7	247
Itel	s32	7	0
Itel	s73	7	0
Itel	s32	7	0
Itel	s32	7	3
Itel	a32f	8	0
Itel	a16	8	0
Itel	a16	8	0
Itel	A16 Plus	8	0

Feasibility of digital contact tracing in low-income settings – pilot trial for a location-based DCT app

Journal: BMC Public Health

Eric Handmann, MD (first author, corresponding author)
Department for Emergency Medicine, University Hospital Leipzig, Leipzig, Germany
Mail: Eric.Handmann@medizin.uni-leipzig.de; ORCID #0000-0001-7584-007X
Sia Wata Camanor, Mosoka P. Fallah, Neima Candy, Davidetta Parker, André Gries, Thomas Grünewald

Itel	p32	8	1301
Itel	s15	9	319
Itel	s15	9	47
Itel	s15	9	240
Samsung	sm g900v	unknown	0
Samsung	J7	unknown	344
Samsung	A300	4	130
Samsung	s3 mini	4	0
Samsung	J5	5	0
Samsung	note5	5	33
Samsung	s6 edge	6	198
Samsung	j7 prime	6	143
Samsung	j7	6	0
Samsung	note4	6	342
Samsung	unknown	6	6
Samsung	note 4	6	0
Samsung	s7	7	387
Samsung	s6	7	210
Samsung	note 5	7	9
Samsung	sm-g920a	7	0
Samsung	s8	8	14
Samsung	j4 plus	8	0
Samsung	s4	8	93
Samsung	s9 plus	8	1
Samsung	s7	8	0
Samsung	s7	8	202
Samsung	Note 4	8	380
Samsung	J3	8	219
Samsung	s7	8	1037
Samsung	Note8	9	1739
Samsung	A7	9	1
Samsung	j8	9	2
Samsung	s8 plus	9	1424
Samsung	s8	9	227
Samsung	j8	9	14
Samsung	a30	9	890
BLU	r1 hd	6	152
BLU	m6 lte	6	3
BLU	c6	8	415
HTC	unknown	7	188
HTC	U11	8	318
Nokia	311	9	825

Feasibility of digital contact tracing in low-income settings – pilot trial for a location-based DCT app

Journal: BMC Public Health

Eric Handmann, MD (first author, corresponding author)
Department for Emergency Medicine, University Hospital Leipzig, Leipzig, Germany
Mail: Eric.Handmann@medizin.uni-leipzig.de; ORCID #0000-0001-7584-007X
Sia Wata Camanor, Mosoka P. Fallah, Neima Candy, Davidetta Parker, André Gries, Thomas Grünewald

LG	ls740	4	55
LG	K8	7	2
Orange	unknown	unknown	0
Orange	rise 32	6	201
One	plus a5010	unknown	23
One	plus a3003	9	24
Vivo	unknown	9	2
Huawei	p smart	8	8
ZTT/ZTE	V6	6	40
ZTT/ZTE	note5	7	686
ZTT/ZTE	unknown	7	160
Motorola	sx4	9	2
Xiaomi	5 plus	7	279
Xiaomi	note 5 pro	9	1045
Alcatel	unknown	7	1257
MobiWire	Halona	5	117
A3	pro	9	1
unknown	unknown	unknown	67
