# Supplementary material for: Feasibility of digital contact tracing in low-income settings – pilot trial for a location-based DCT app
Source: BMC Public Health. 2023 Jan 21;23:146. doi: 10.1186/s12889-022-14888-x (PMC9859743; doi:10.1186/s12889-022-14888-x)
Supplement: Supplementary file 5 — Additional file 5. Infectet # 7 - Logged GPS coordinates (yellow tags) of Infected #7 on day 12 were analyzed to check for possible false negative results. There were no aCT hits despite 17:15 hours tracking, due to the fact that Infected #7 was in the hospital only at nighttime and large parts of the tracked time was spend remote from the hospital, where he did not meet other participants. Furthermore a big variance of GPS locations is displayed. [file 12889_2022_14888_MOESM5_ESM.pdf]

Feasibility of digital contact tracing in low-income settings – pilot trial for a location-based DCT app

Journal: BMC Public Health

Eric Handmann, MD (first author, corresponding author)

Department for Emergency Medicine, University Hospital Leipzig, Leipzig, Germany

Mail: Eric.Handmann@medizin.uni-leipzig.de; ORCID #0000-0001-7584-007X

Sia Wata Camanor, Mosoka P. Fallah, Neima Candy, Davidetta Parker, André Gries, Thomas Grünewald

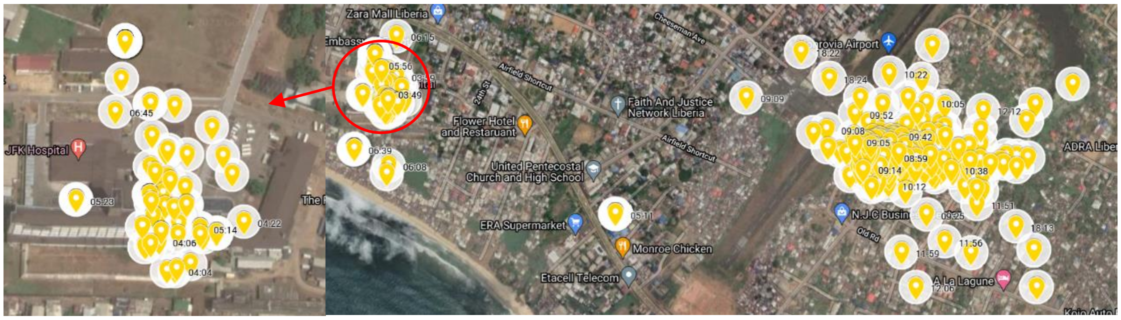

AF 5 Logged GPS coordinates (yellow tags) of Infected #7 on day 12 *were analyzed to check for possible false negative results*. There were no aCT hits despite 17:15 hours tracking, due to the fact that Infected #7 was in the hospital only at nighttime and large parts of the tracked time was spend remote from the hospital, where he did not meet other participants. Furthermore a big variance of GPS locations is displayed.
