# Supplementary material for: Feasibility of digital contact tracing in low-income settings – pilot trial for a location-based DCT app
Source: BMC Public Health. 2023 Jan 21;23:146. doi: 10.1186/s12889-022-14888-x (PMC9859743; doi:10.1186/s12889-022-14888-x)
Supplement: Supplementary file 6 — Additional file 6. Overall acceptance – Survey results on overall acceptance of the DCT. [file 12889_2022_14888_MOESM6_ESM.pdf]

Feasibility of digital contact tracing in low-income settings – pilot trial for a location-based DCT app

Journal: BMC Public Health

Eric Handmann, MD (first author, corresponding author)
Department for Emergency Medicine, University Hospital Leipzig, Leipzig, Germany
Mail: Eric.Handmann@medizin.uni-leipzig.de; ORCID #0000-0001-7584-007X
Sia Wata Camanor, Mosoka P. Fallah, Neima Candy, Davidetta Parker, André Gries, Thomas Grünewald

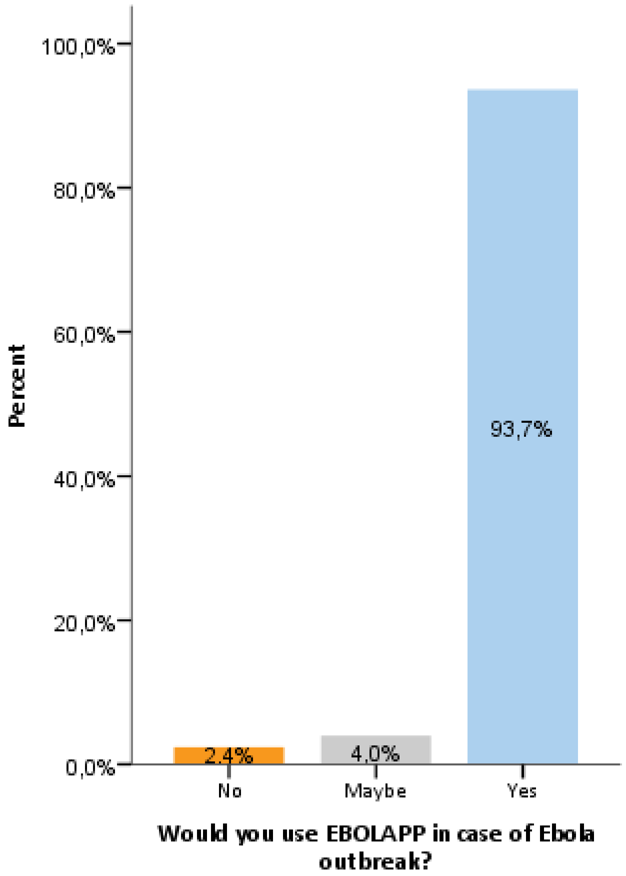

AF 6 Survey results on overall acceptance of the DCT
